# Respiratory Syndrome Coronavirus Infections: Possible Mechanisms of Neurological Implications—A Systematic Review

**DOI:** 10.3389/fneur.2020.00864

**Published:** 2020-08-21

**Authors:** Gilmara Gomes de Assis, Eugenia Murawska-Cialowicz, Pawel Cieszczyk, Eugene V. Gasanov

**Affiliations:** ^1^Department of Molecular Biology, Gdansk University of Physical Education and Sports, Gdansk, Poland; ^2^Department of Physiology and Biochemstry, University School of Physical Education in Wrocław, Wrocław, Poland; ^3^Laboratory of Neurodegeneration, International Institute of Molecular and Cell Biology in Warsaw, Warsaw, Poland

**Keywords:** Covid-19, SARS-CoV2, coronavirus, neurovirulence, pathogenicity

## Abstract

Within the context of the worst pandemic of the century—Covid-19—which emerged in China and has spread across the entire globe over the last 6 months, increased knowledge about viral behavior that be prognostic is crucial. Following the patterns of other coronaviruses (CoVs), particularly those infecting the respiratory tract, neurological manifestations have been reported in patients with Covid-19. Such manifestations highlight the neurovirulence of this severe acute respiratory syndrome (SARS)-CoV2. In order to collect all available information on the implications and mechanisms of infections by respiratory CoVs, a systematic review was designed following the PRISMA protocol. The following PICO strategy (patient, problem, or population; intervention; comparison, control, or comparator; outcomes) was adopted: P included healthy individuals, patients, and animal models susceptible to human-specific viruses; I included molecular, cell culture, and comparative experimental studies; C included healthy, diseased, and immunized conditions; and O represented the virulence and pathogenicity of respiratory CoVs and their effects on the central nervous system (CNS). Searches were conducted in PubMed databases from March 30 to April 1, 2020. Results indicate the involvement of the CNS in infections with various CoVs. Infection typically begins in the airway epithelia with subsequent alveolar involvement, and the virus then spreads to the CNS via neuronal contacts with the recruitment of axonal transport. Neuronal infection and regulated cell death are the main factors causing a generalized encephalitis.

## Introduction

Viral neurotropism with the potential for acute and/or chronic consequences to the central nervous system (CNS) has been identified since the late 1950s with findings of the involvement of a murine hepatitis virus (MHV) in encephalomyelitides in humans (1, 2). The name coronavirus (CoV) emerged in a small note published in 1968 by a group of virologists who published their papers in the *Nature* journal. They showed that the viral particles are more or less rounded, although they noted a certain degree of polymorphism, with a fringe of projections, which are rounded or petal-shaped, rather than sharp or pointed. This appearance, resembling the solar corona, inspired the name that was adopted for MHV and several viruses recovered from humans at the time (3).

Coronaviruses (in this study referred only to those human-specific infectious) enclose a group of eukaryotic spherical RNA viruses, which infect animals and humans by fecal-oral and respiratory routes, as well as mechanical transmission. Several species of CoVs have been transmitted among humans, causing epidemics of various proportions. In just 6 months, the current Covid-19 (Coronavirus disease-2019) pandemic, caused by infection with the respiratory CoV named “SARS-CoV2” (severe acute respiratory syndrome coronavirus), has become the greatest global public health and economic crisis seen in generations. Most recently, reports of medical doctors operating on the front line of the ongoing pandemic suggest the incidence of neuropathological manifestations associated with SARS-CoV2 infections (4, 5).

Similarities between SARS-CoV2 and other respiratory CoVs have also been reported. For instance, SARS-CoV2, like other respiratory CoVs that infect humans, binds to the angiotensin-converting enzyme 2 (ACE2) receptor, which is widely distributed throughout the respiratory tract epithelium, lung parenchyma, and gastrointestinal tract (6). Respiratory distress in patients with Covid-19 may be the result of both pulmonary inflammatory structural damage, as well as damage caused in the respiratory centers of the brain (7). However, reports of such distress require further investigation on the possible mechanisms of neurovirulence associated with CoV infections of the respiratory system.

Furthermore, beyond the pulmonary, renal, cardiac, and circulatory damage that can prove to be fatal in patients with Covid-19, a dominant cerebral involvement with the potential to cause cerebral edema can be a leading cause of death, long before systemic homeostatic dysregulation (8). Thus, a critical view of the scientific evidence of human infections with a wider spectrum of respiratory CoVs is necessary to elucidate the possible mechanisms of SARS-CoV2 interactions with the nervous system.

In order to substantiate possible implications of the Covid-19 pandemic for neurology and gain a better understanding of the virulence and pathogenicity of SARS-CoV2, we performed a systematic review. We collected all available data at present in the PubMed databases on viruses of the CoV family, which cause respiratory infections in humans and have implications for the nervous system.

## Methods

In order to retrieve all available data on respiratory infections with CoVs that have an effect on the nervous system, a search strategy was conducted in the Medical Subject Headings PubMed platform from March 30 to April 1, 2020, using the following combinations of terms: neurons vs. coronavirus; neural stem cells vs. coronavirus; nervous system vs. coronavirus; SARS virus vs. neurons; SARS virus vs. neural stem cells; and SARS virus vs. nervous system. A total of 484 papers were retrieved and downloaded in the Mendeley software and duplicates were removed. Studies were subjected to double-blinded screening by their titles and abstracts for inclusion criteria regarding the following PICO strategy:

P (patient, problem, or population): healthy individuals and patients with neurological disorders;
I (intervention): immunology and molecular tests, cell cultures, and comparative experimental studies of respiratory CoV in humans;
C (comparison, control, or comparator): healthy vs. diseased conditions, health vs. immunized conditions;
O (outcomes): virulence and pathogenicity of the virus in the CNS.

**Figure 1:**
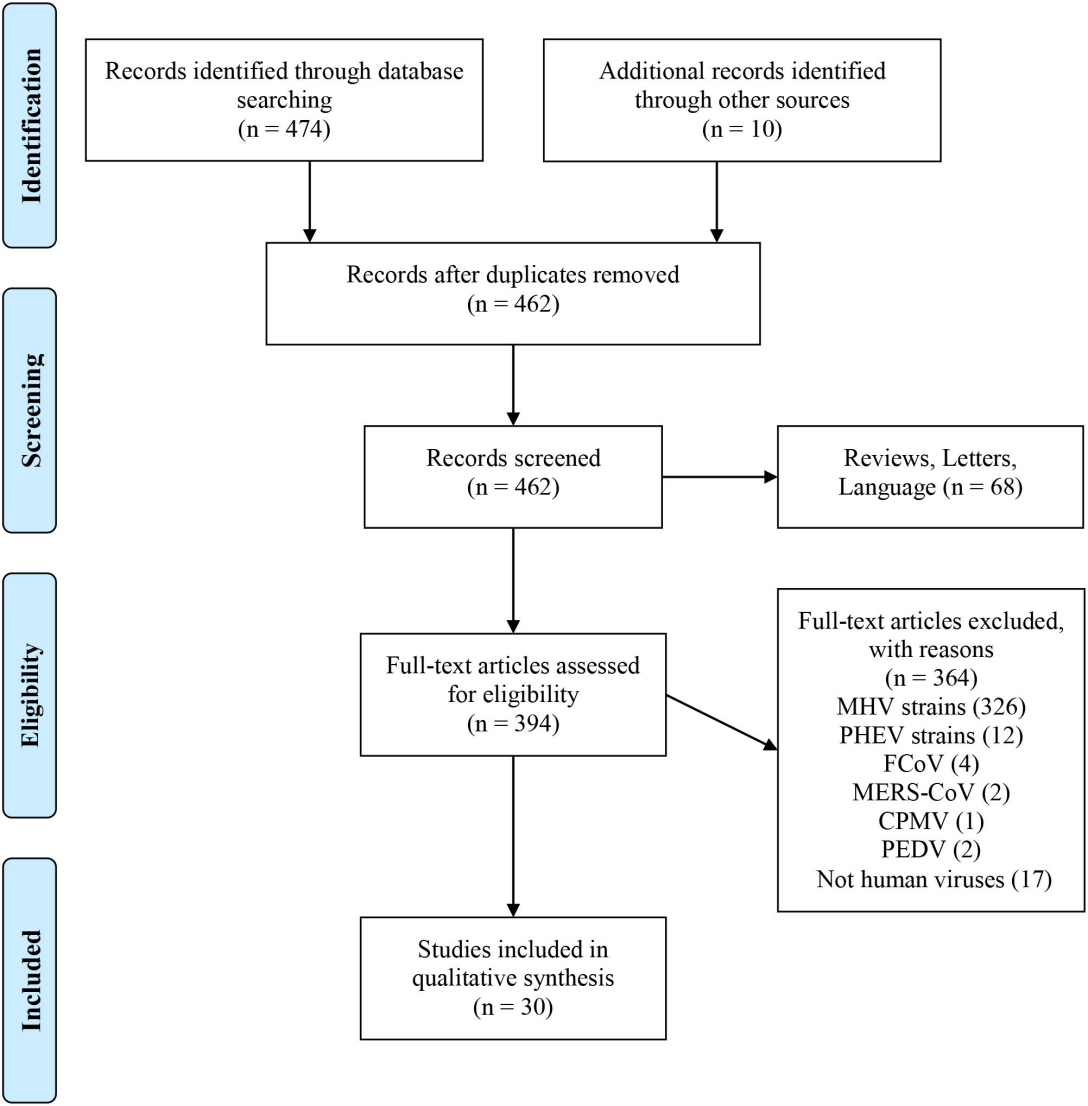
PubMed data extraction flow chart. From Moher et al. (9). For more information, visit www.prisma-statement.org.

Reviews, letters, commentaries, non-interventional studies, articles not written in English, and animal studies without a focus on human retroviruses were all excluded (Figure 1).

**Figure 2:**
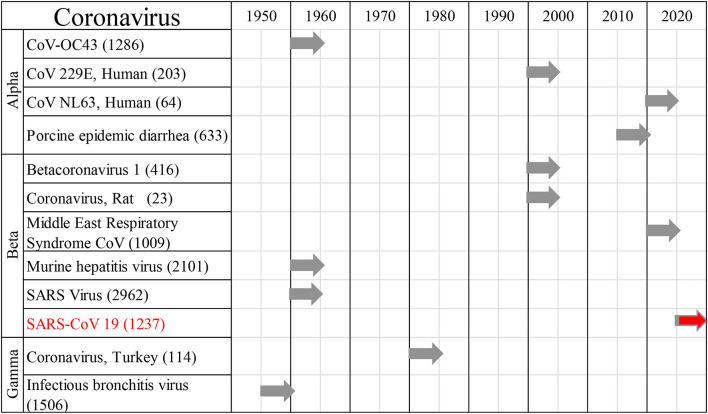
Human coronaviral infections. First reports on the different types of Coronaviruses infecting humans throughout history, and the number of published studies.

All reports of human CoV infections found at this stage were displayed on a timeline (Figure 2). A total of 30 articles were included for synthesis without meta-analysis (10). This review followed the PRISMA (preferred reporting items for systematic reviews and meta-analyses) protocol. The studies' findings are summarized in Table 1.

**Table 1:** Studies and main findings.

**Authors and Title**	**Virus**	**Model**	**Main findings**
(11) “From Patients with Upper Respiratory Tract Disease”	IBV-like virus strains	Suckling mouse and tracheal cell culture	Two of the six “IBV-like” strains caused an encephalitic syndrome in inoculated mice
(12) “Some Characteristics of Hemagglutination of Certain Strains of ‘IBV-like’ Virus”	IBV-like	Mouse brain harvests, human, chicken, mouse, rat rhesus and guinea pig cells, erythrocytes	Human cells were agglutinated without spontaneous elution at an optimal hemagglutination temperature
(13) “Differential Susceptibility of Cultured Neural Cells to the Human Coronavirus OC43”	CoV-OC43	Neural cell cultures	Human embryo brain cells, including astrocytes, were susceptible to OC43 infection but did not produce infectious virus
(14) “Interferon γ Potentiates Human Coronavirus OC43 Infection of Neuronal Cells by Modulation of HLA Class I Expression”	CoV-229E CoV-OC43	Human cortical neuron, neuroblastoma, and diploid lung cell lines	CoVs were able to replicate in neurons
(15) “Infection of Primary Cultures of Human Neural Cells by Human Coronaviruses 229E and OC43”	CoV-229E CoV-OC43	Human neural cell lines	Microglial cells did not produce infectious progeny viruses after CoV-OC43 infection
(16) “Involvement of Aminopeptidase N (CD13) in Infection of Human Neural Cells by Human Coronavirus 229”	CoV-229E	Human embryonic lung and neural cell lines	Expression of aminopeptidase-N receptor in neurons, astrocytes, and oligodendrocytes might explain their susceptibility to CoV-229E infection
(17) “Persistent Infection of Human Oligodendrocytic and Neuroglial Cell Lines by Human Coronavirus 229E”	CoV-229E	Human neural cell lines	Oligodendrocytic and neuroglioma cell lines also sustained a persistent viral infection
(17) “Neuroinvasion by Human Respiratory Coronaviruses”	CoV-229E CoV-OC43	Human brain autopsy samples–various neurological diseases	Higher prevalence of CoV-OC43 in patients with MS compared with the controls
(18) “Activation of Glial Cells by Human Coronavirus OC43 Infection”	CoV-OC43	Immortalized human microglial cells and human astrocyte cell line	CoV infection of glial cells may be indirectly associated with CNS pathologies
(19) “Coronaviruses in Brain Tissue from Patients with Multiple Sclerosis”	CoV-229E CoV-OC43	Brain tissue from patients with MS	Evidence for chronic infection with CoV-229E or OC43 in the brain tissue of patients with MS or controls has not been found
(20) “Vacuolating Encephalitis in Mice Infected by Human Coronavirus OC43”	CoV-OC43	Mice	Damage to the CNS was not immunologically mediated and the microglial reactivity was a consequence of neural infection
(21) “Possible Central Nervous System Infection by SARS Coronavirus”	SARS-CoV	32-years-old woman, week 26 of pregnancy, previously in good health	Generalized tonic-clonic convulsions and positive SARS-CoV in cerebral spinal fluid suggested infection of the CNS with SARS-CoV
(22) “Human Respiratory Coronavirus OC43: Genetic Stability and Neuroinvasion”	CoV-OC43	Cell culture	Described the complete genome sequence of CoV-OC43 strains
(23) “Mechanisms of Host Defense Following Severe Acute Respiratory Syndrome coronavirus (SARS-CoV) Pulmonary Infection of Mice”	SARS-CoV	Mice	SARS-CoV generated a transient non-fatal systemic infection in the lungs which was disseminated to the brain
(24) “Multiple organ infection and the pathogenesis of SARS”	SARS-CoV	Brain tissue, full autopsy of 39-years-old patient with SARS	Neuroinvasion by SARS-CoV, evidenced by viral morphology, genetic identification, and the viral antigen (N protein) found in the brain
(25) “Susceptibility of Human and Rat Neural Cell Lines to Infection by SARS Coronavirus”	SARS-CoV	Human oligodendroglioma, Rat glioma, Human intestine, Canine kidney, and Rabbit kidney cell lines	Human and rat neural cells were susceptible to SARS-CoV infection, with no apparent cytopathic effects
(26) “Murine Encephalitis Caused by HCoV-OC43, a Human Coronavirus with Broad Species Specificity, Is Partly Immune-Mediated”	CoV-OC43	Mice	Rapidly increase in virulence after passage in the brain is likely to occur via selection of mutations in the S-glycoprotein
(27) “Human Coronavirus OC43 Infection Induces Chronic Encephalitis Leading to Disabilities in BALB/C Mice”	CoV-OC43	Mice neural cell lines	Results support the theory that CoV-OC43 has a preferential tropism for neurons
(28) “Lethal Infection of K18-HACE2 Mice Infected with Severe Acute Respiratory Syndrome Coronavirus”	SARS-CoV	Mice transgenic for ACE2 receptor	Transgenic mice developed a rapidly lethal infection that spread to the brain, after intranasal inoculation with SARS-CoV
(29) “Severe Acute Respiratory Syndrome Coronavirus Infection of Mice Transgenic for the Human Angiotensin-Converting Enzyme 2 Virus Receptor”	SARS-CoV	Mice transgenic for ACE2 receptor	Lungs and brain were the major sites of viral replication, particularly in transgenic mice
(30) “Long-Term Human Coronavirus-Myelin Cross-Reactive T-Cell Clones Derived from Multiple Sclerosis Patients”	CoV-229E CoV-OC43	T-cell clones (TCC) of patients with MS	TCC from the blood of patents with MS could be activated by either viral or myelin antigen and sometimes by both
(31) “Pathological Changes in Masked Palm Civets Experimentally Infected by Severe Acute Respiratory Syndrome (SARS) Coronavirus”	SARS-CoV	Masked palm civets	SARS-CoV caused a multi-organ pathology in civets similar to that observed in human patients with SARS
(32) “Severe Acute Respiratory Syndrome Coronavirus Infection Causes Neuronal Death in the Absence of Encephalitis in Mice Transgenic for Human ACE2”	SARS-CoV	Mice transgenic for ACE2 receptor	Neurons are highly susceptible targets for SARS-CoV infection and the absence of cell receptors prevents severe murine brain disease
(33) “Neuroprotective Effect of Apolipoprotein D against Human Coronavirus OC43-Induced Encephalitis in Mice”	CoV-OC43	Human apolipoprotein D(apoD) transgenic mice	Overexpression of apoD in neurons resulted in an increased number of survivors to CoV-OC43 infection
(34) “Glutamate Excitotoxicity is Involved in the Induction of Paralysis in Mice after Infection by a Human Coronavirus with a Single Point Mutation in Its Spike Protein”	CoV-OC43	Mice	The AMPA receptor antagonist led to reduced microglial activation, which was believed to improve the regulation of CNS glutamate homeostasis
(35) “Human Coronavirus-Induced Neuronal Programmed Cell Death Is Cyclophilin D Dependent and Potentially Caspase Dispensable”	CoV-OC43	Human neuronal cell lines	Mitochondrial apoptosis-inducing factor and cyclophilin D appears to be pivotal in CoV-OC43-induced programmed cell death, while caspases do not appear to be essential
(36) “Novel Treatment with Neuroprotective and Antiviral Properties against a Neuroinvasive Human Respiratory Virus”	CoV-OC43	Mice	Memantine improved clinical scores related to the motor disabilities and attenuated mortality rates in virus-infected mice
(37) “Pivotal Role of Receptor-Interacting Protein Kinase 1 and Mixed Lineage Kinase Domain-Like in Neuronal Cell Death Induced by the Human Neuroinvasive Coronavirus OC43”	CoV-OC43	Human neuroblastoma cell lines and mice	A CoV-OC43 variant, harboring two-point mutations in the S-glycoprotein (S2) was more neurovirulent than the CoV-OC43 in mice and induced more cell death in murine and human neuronal cells
(38) “The OC43 Human Coronavirus Envelope Protein Is Critical for Infectious Virus Production and Propagation in Neuronal Cells and Is a Determinant of Neurovirulence and CNS Pathology”	CoV-OC43	Human neuronal cell lines and mouse neuronal cell lines	CoV-OC43 envelope protein is critical for the production of infectious virions
(39) “Axonal Transport Enables Neuron-to-Neuron Propagation of Human Coronavirus OC43”	CoV-OC43	Human neuronal cell lines and mice	Both passive diffusion of released viral particles and axonal transport are valid propagation strategies used by the virus

*CoV, coronavirus; CNS, central nervous system; MS, multiple sclerosis; ACE2, angiotensin-converting enzyme 2; AMPA, 2-amino-3-(5-methyl-3-oxo-1,2-oxazol-4-yl) propanoic acid*.

## Results

Six strains of infectious bronchitis virus (IBV) were detected in embryonic tracheal organ cultures from patients with colds. McIntosh et al. (11) demonstrated that two of these “IBV-like” strains were able to grow in newborn inoculated mice, and caused an encephalitic syndrome. Complement-fixation tests of human convalescent sera and the specific mouse immune sera were homologous to the brain suspensions from affected mice. The strains were shown to be identical with each other and distinct from IBV and strain CoV-229E (another respiratory CoV, morphologically similar to IBV). Kaye and Dowdle (12) discovered two strains of IBV-like CoVs and named them “OC38” and “OC43.”

Pearson and Mims (13) investigated selective cell vulnerability to CoV-OC43 infection. Using cell-type-specific markers and neural cultures derived from various areas of the CNS, they showed that neurons from the dorsal root ganglia produced both viral antigen and infectious virus, while astrocytes and fibroblasts produced only viral antigen, and oligodendrocytes produced neither the infectious virus nor viral antigen. Human embryo brain cells, including astrocytes, are susceptible to OC43 infection but production of infectious virus has not been reported.

Collins (14) showed that cortical neuronal cells support the replication of both CoV-229E and CoV-OC43 serotypes. Using a human cerebral neuron cell line, the results of that study showed that neurons, which express the aminopeptidase-N receptor (CD13) for CoV-229E at nerve synapses, are much more susceptible to direct infection by CoV-229E than by CoV-OC43. Both viruses induced the synthesis of viral antigens.

Using antibodies to CoV-229E and CoV-OC43 viruses, and cell markers in human neural primary cultures cells, Bonavia et al. (15) was able to demonstrate viral neuroinvasion in fetal astrocytes, and in adult microglia and astrocytes, by the strain OC43. Furthermore, RNA amplification also confirmed infection of fetal astrocytes, adult microglia, and a mixed culture of adult oligodendrocytes and astrocytes with the strain 229E. In this study, infectious virus was released only from fetal astrocytes, with higher titers for CoV-OC43.

Lachance et al. (16) tested whether CD13 is utilized as a receptor for CoV-229E infection inhuman neural cell lines. They proved that CD13 expression on the surfaces of various neuronal and glial cell lines, which are susceptible to CoV-229E infection, correlate with the level of viral attachments. They also showed that CD13 is expressed and serves as a receptor for CoV-229E infection in neuronal and glial cells.

Arbour et al. (17) provided evidence of CoV-229E neurotropism, and possible viral persistence in the CNS by showing that astrocytoma, neuroblastoma, neuroglioma, and oligodendrocytic cell lines are all susceptible to infection by CoV-229E. The oligodendrocytic and neuroglioma lines sustained a persistent viral infection, which was monitored by detection of the viral antigen and infectious viral progeny.

Arbour et al. (40) characterized CoV RNA in a large panel of human brain autopsy samples. Amplified CoV-229E and CoV-OC43 strains from the samples of donors with various neurological diseases 39 with multiple sclerosis (MS), 26 with other neurological diseases, and 25 controls) reported that 44% (40 of 90) of donors were positive for CoV-229E and 23% (21 of 90) were positive for CoV-OC43. A higher prevalence of CoV-OC43 was noted in patients with MS (35.9%; 14 of 39) than in the controls (13.7%; 7 of 51). Viral RNA was found in brain parenchyma, but outside of the blood vessels.

Evidence of a CoV-induced MS-like in rodents, which plays a role in the inflammatory system, led Edwards et al. (18) to analyze the expression of cytokines and chemokines in CoV-OC43-infected human astrocytes and immortalized microglial cell lines. An up-regulation of IL-6, TNF-a, and MCP-1 mRNA expression was observed in the astrocytes infected with CoV-OC43. The virus also modulated the activity of matrix metalloproteinases-2 and -9 and augmented nitric oxide production in microglial cells.

Brain tissue samples from 25 patients with MS and 36 controls were tested for the prevalence of CoV (19). Four PCR assays with primers specific for the N-protein gene of CoV-229E and three PCR assays specific for the nucleocapsid protein gene of CoV-OC43 were performed. Some sporadic positive PCR assays were observed in both patients and controls. Results were not reproducible and no significant difference was reported in the proportion of positive signals from the patients with MS compared with the controls.

Jacomy and Talbot (20) developed an experimental model of CoV-OC43-inoculated mice and characterized the neurotropic properties of the virus. The virus led to a generalized infection of the entire CNS. The acute infection targeted neuronal cells, which underwent vacuolation and degeneration, during strong microglial reactivity and inflammatory reactions. Damage to the CNS was not immunologically mediated and the microglial reactivity was instead, a consequence of direct virus-mediated neuronal injury.

Lau et al. (21) reported a case of a 32-years-old healthy woman in week 26 of pregnancy, who was admitted to the hospital with myalgia, fever, chills, and rigor for 2 days. She had tested positive for the “new” severe acute respiratory syndrome CoV (SARS-CoV) in cerebrospinal fluid collected from day 22, after generalized tonic-clonic convulsions with a loss of consciousness.

St-Jean et al. (22) uncovered six mutations scattered throughout the CoV-OC43 genome giving rise to two amino acid substitutions. The two CoV-CO43 variants were able to reach the CNS after intranasal inoculation in mice. The stability of the virus in the environment was highlighted, when the two variants were isolated from cells, 40 years apart. Genomes of the two CoV-OC43 variants displayed 71, 53.1, and 51.2% identity with MHV A59, the SARS-CoV Tor2 strain, and CoV-229E, respectively. Furthermore, CoV-OC43 has well-conserved motifs, like the genome sequence of the Tor2 strain of SARS-CoV, suggesting that CoV-OC43 and SARS-CoV may share several important functional properties.

Glass et al. (23) produced a model of SARS-CoV infection in mice. Intranasally introduced virus replicated transiently to high levels in the lungs, with a peak on day 3 post-infection and clearance by day 9. Viral RNA was localized to the bronchial and bronchiolar epithelium and mRNA expression for the ACE2 receptor was detected in the lung, following infection. The expression of the proinflammatory chemokine genes, *CCL2, CCL3, CCL5, CXCL9*, and *CXCL10*, and receptor genes (especially *CXCR3*) was up-regulated in the lungs with differential kinetics. However, T-cell cytokine mRNAs (Th1 and Th2) were not detectable. Minimal local accumulation of leukocytes was observed without obvious clinical signs of pulmonary dysfunction. Infection also spread from the lungs to the brain without leukocyte accumulation. Mice showed normal clearance of the virus.

Gu et al. (24) isolated a SARS-CoV strain from brain autopsies. Fifteen cytokines and chemokines were detected in the blood of a patient. Amplifications of SARS-CoV specific fragments from the brain tissue of eight patients confirmed the presence of the virus. Pathologic examination showed necrosis of neuronal cells and extensive hyperplasia of gliocytes. The *CXCL9* gene was expressed in gliocytes and the infiltration of monocytes/macrophages and T lymphocytes was observed in the brain mesenchyme.

Both *CXCL10* and *CXCL9* expression levels were highly elevated in the blood, although the levels of other cytokines and chemokines were close to normal. Chest radiographs indicated that the pathologic change in the brain was independent of pulmonary superinfection. Neuroinvasion by SARS-CoV was confirmed by viral morphology observed under the microscope, as well as genetic identification, and the presence of viral antigen (N protein) in the brain.

Yamashita et al. (25) showed that SARS-CoV infection in human neural cells can yield viral infectivity of 10^2−5^ per mL, with no apparent cytopathic effects. Infection of the intestinal cell line “CaCo-2” also induced no apparent cytopathic effects, with the production of lower levels of the virus. The SARS-CoV receptor, ACE2, was expressed at higher levels on CaCo-2 cells, but at undetectable levels in neural cells.

Butler et al. (26) showed that CoV-OC43 is highly virulent in the suckling mouse brain and can cause a uniformly fatal encephalitis in adult mice. A spike glycoprotein (S-glycoprotein) on the surface of the CoV-OC43 membrane was revealed as a major virulence factor in CoV infections. The ability to rapidly gain virulence after passage in the murine brain is likely to occur via the selection of mutations in the S-glycoprotein, which optimize both binding and entry to target cells. Three changes in the CoV-OC43 S-glycoprotein, in the domain of the protein responsible for binding to host cells, correlated with enhanced neurovirulence in the model. Such adaptability has facilitated the isolation of CoV-OC43 variants with markedly differing abilities to infect animals and tissue culture cells. This adaptability also appears to be a mechanism by which CoV-OC43 and SARS-CoV can readily adapt to growth in cells from heterologous species.

Jacomy et al. (27) used primary cell cultures to determine the susceptibility of each type of neural cell toCoV-OC43 infection. They showed that neurons are the target cells undergoing degeneration during infection, in part due to apoptosis. Intracerebral inoculation with CoV-OC43 in susceptible mice led to an acute encephalitis, with neuronal cell death by necrosis and apoptosis. Infectious viral particles were apparently cleared from surviving animals, whereas viral RNA persisted for several months. After the acute encephalitis, some of the surviving animals presented an abnormal limb-clasping reflex and reduced motor activity starting several months post-infection.

McCray et al. (28) produced transgenic mice that expressed the human ACE2 receptor in the airway and other epithelia. Mice developed a rapidly lethal infection after intranasal inoculation with SARS-CoV. Infection began in the airway epithelia, with subsequent alveolar involvement, and extrapulmonary virus spread to the brain. Furthermore, infection resulted in the infiltration of macrophages and lymphocytes into the lungs and an upregulation of proinflammatory cytokines and chemokines in both the lungs and brain.

In the study Tseng et al. (29), the best model of transgenic mice expressing the human ACE2 receptor showed clinical manifestations within 8 days post-intranasal SARS-CoV infection. High viral titers were detected in the lungs and brains on days 1 and 3. Inflammatory mediators in these tissues coincided with the high levels of viral replication. Lower viral titers were detected in the blood. Infected non-transgenic mice survived, without showing signs of clinical illness. Extensive CNS involvement likely determines whether the animal dies, rather than the presence of viral pneumonia. Mouse lineages, in which the transgene expression is considerably less abundant than it was in this model, showed that viral replication is largely restricted to the lungs but not the brain.

Boucher et al. (30), after reporting the isolation of CoV-229E/myelin basic protein (MBP) cross-reactive T cell lines (TCL) in patients with MS, checked for antigenic cross-reactivity. A total of 155 long-term T-cell clones (TCC) were derived from 32 patients with MS by *in vitro* selection against MBP, proteolipid protein, or CoV (strains 229E and OC43). Overall results showed that 114 TCC were virus-specific, 31 were specific for the myelin antigen, and 10 were CoV/myelin cross-reactive. Additionally, 28 virus-specific TCC and seven myelin-specific TCC were obtained from six healthy donors. The TCC derived from the blood of patients with MS could have been activated by either viral or myelin antigen, and sometimes by both.

Xiao et al. (31) elucidated the histopathological changes induced by SARS-CoV in an infected civet (*Paguma larvata*) model. *In-situ* hybridization detected viral RNA in the animal's lung, small intestine, and brain alone. Apoptosis detection within the brain showed evidence of neuronal degeneration and mild neuronophagia from days 3 to 13 after infection. In two cases, mild edema was also observed around the small veins and nerve cells. No microscopic changes were observed in these tissues after 13 days. Furthermore, no evidence of apoptosis was observed within the myocardium. Those results indicated that SARS-CoV can cause a multi-organ pathology in civets, similar to that observed in human patients with SARS.

Netland et al. (32) experimented with transgenic mice and the ACE2 receptor. They showed that the virus enters the animal's brain primarily via the olfactory bulb and the infection results in a rapid trans-neuronal spread to other connected areas of the brain. Extensive neuronal infection was the main cause of death, considering that intracranial inoculation with low doses of the virus results in a uniformly lethal disease, even when little infection is detected in the lungs. Death likely results from dysfunction and/or death of infected neurons, especially those located in cardiorespiratory centers in the medulla. The SARS-CoV induced minimal cellular infiltration in the brain. The results of that study confirmed that neurons are highly susceptible targets for SARS-CoV infection, in which only the absence of the cell receptor prevented severe murine brain disease.

Do Carmo et al. (33) showed that apolipoprotein D (apoD) expression is regulated during acute encephalitis induced by CoV-OC43 infection. The upregulation of human apoD expression in transgenic mice coincided with glial activation. Both apoD expression and glial activity returned to normal levels when the virus was cleared. Overexpression of apoD in the neurons of mice resulted in a 3-fold increase in the number of mice surviving the CoV-OC43 infection. The overexpression of apoD also correlated with up-regulated glial activation, a limited innate immune response (associated with cytokines and chemokines), and T-cell infiltration into infected brains.

Brison et al. (34) showed that a single point mutation in the S-glycoprotein of CoV-OC43, acquired during viral persistence in human neural cells, led to a hindlimb paralytic disease in mice. In the S-glycoprotein mutant mice, the inhibition of glutamate excitotoxicity, using a 2-amino-3-(5-methyl-3-oxo-1,2-oxazol-4-yl) propanoic acid (AMPA) receptor antagonist, improved clinical scores associated with paralysis and motor disabilities. Glutamatergic inhibition also protected the CNS from neuronal dysfunction, as illustrated by the restoration of phosphorylation of neurofilaments. Expression of the glial glutamate transporter (GLT-1), responsible for glutamate homeostasis, was down-regulated following infection, while the AMPA receptor antagonist restored GLT-1 steady-state expression levels. Treatment with the AMPA receptor antagonist led to reduced microglial activation, which was believed to improve the regulation of CNS glutamate homeostasis.

After observing that CoV-OC43 infection of neurons activates the unfolded-protein response and caspase-3, and induces cell death with involvement of the S-glycoprotein, Favreau et al. (35) elucidated possible mechanisms of cell death following CoV-OC43 infection. They reported a more neurovirulent and cytotoxic CoV-OC43 variant, harboring two-point mutations in the S-glycoprotein (S2) in human neuronal cells. Caspase-3 and -9 were both activated after infection, but caspase inhibitors neither reduced nor delayed virus-induced cell death. The proapoptotic proteins, BAX and cytochrome c (CytC), and the apoptosis-inducing factor were re-localized toward the mitochondria, cytosol, and nucleus, respectively, after infection with the variants of both viruses. Neuronal cells treated with cyclosporine (an inhibitor of the mitochondrial permeabilization transition pore), or knocked down for cyclophilin D (a protein known for regulating mitochondrial function) were completely protected from CoV-OC43-induced neuronal death. However, knockdown of S2 in infected cells had a moderate effect on programmed cell death The results supported the theory that mitochondrial apoptosis-inducing factor and cyclophilin D are central to CoV-OC43-induced cell death, while caspases do not appear to be essential.

Brison et al. (36) demonstrated that glutamate recycling, via GLT-1 and glutamine synthetase, is central to the dysregulation of glutamate homeostasis and development of motor dysfunctions and paralytic disease in CoV-OC43-infected mice. Furthermore, memantine (an N-methyl-D-aspartate receptor antagonist, widely used in the treatment of neurological diseases) improved clinical scores related to motor disabilities, by partially restoring physiological neurofilament phosphorylation in virus-infected mice and attenuating mortality rates. Reduced CoV-OC43 replication was observed in the CNS in a dose-dependent manner.

Meessen-Pinard et al. (37) verified whether knockdown of BAX (BCL2-associated X protein) or RIP1 (a key regulator of necroptosis) altered the percentage of neuronal cell death following CoV-OC43 infection. They reported that BAX-dependent apoptosis did not play a role in regulated cell death following infection, once the inhibition of BAX expression, mediated by RNA interference, did not confer cellular protection against the cell death process. Both RIP1 and mixed lineage kinase domain-like (MLKL) were involved in neuronal cell death, as RIP1 knockdown and chemical inhibition of MLKL increased cell survival after infection. The results indicated that RIP1 and MLKL contribute to necroptotic cell death, following CoV-OC43 infection, to limit viral replication. This regulated cell death can lead to neuronal loss and accentuate the neuroinflammatory process, reflecting the severity of neuropathogenesis.

Stodola et al. (38) used recombinant viruses, either devoid of the structural envelope (E) protein or harboring mutations in the putative transmembrane domain or PDZ-binding motif. They demonstrated that with CoV-OC43 infection of cell lines from the human CNS and mouse CNS, the E protein is critical for efficient and optimal viral replication and propagation, and therefore, neurovirulence.

Dubé et al. (39) showed a route of neuropropagation from the nasal cavity to the olfactory bulb and piriform cortex, and then to the brain stem in mice. A neuron-to-neuron propagation was identified as one underlying mode of viral spread in cell culture. Both passive diffusion of released viral particles and axonal transport were propagation strategies used by the virus. The presence of viral platforms with static dynamism was consistent with the viral assembly sites revealed along the axons. The CoV-OC43 modes of propagation might be modulated by selected CoV-OC43 proteins and axonal transport.

## Discussion

**Figure 3:**
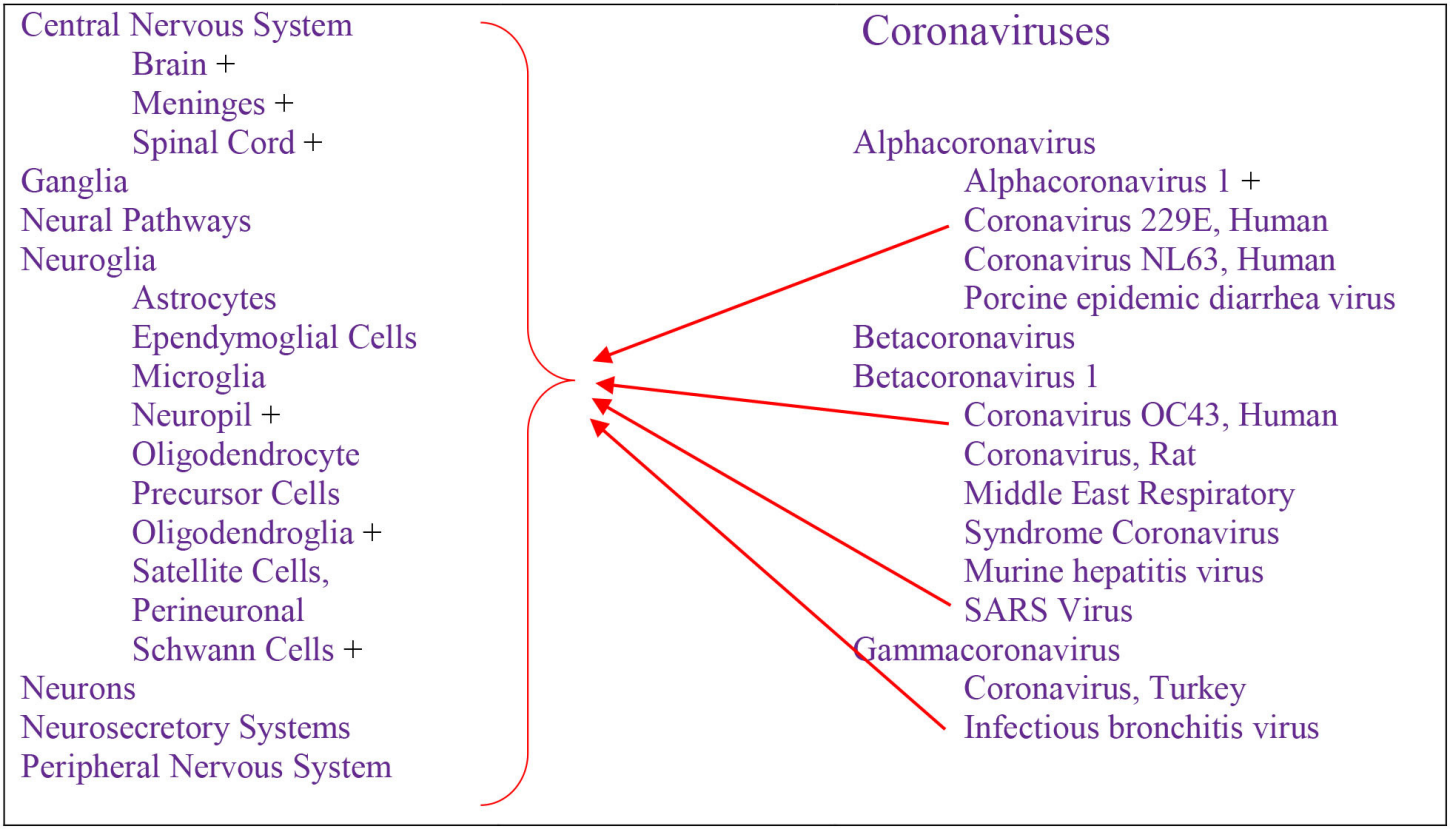
Respiratory-Neurovirulent Coronaviruses detected in human—PubMed source.

Cumulative data indicate that respiratory infection with different species of CoV can evolve to CNS disturbances, sequelae, and possibly chronic disease (Figure 3). Respiratory CoVs have been identified for more than eight decades, with the most devastating scenario created by the current Covid-19 pandemic following the SARS-CoV2 outbreak. Search results showed that the last 6 months of the pandemic has already produced more speculation than the entire body of scientific literature on any other CoV. Historically, IBV-like, CoV-OC43, CoV-229E, and SARS-CoV have been shown to interact with the CNS (Figure 2).

Reports of respiratory infections with CoVs among several studies show similar levels of neuropathogenicity. The binding of these viruses to the ACE2 receptor is putative for viral neuroinvasion, even though neuronal viral infection may be mediated by the CD13 receptor. IN addition, CoV-229E, CoV-OC43, and SARS-CoV have been shown to have the capacity to infect neurons, astrocytes, glial cells, and fibroblasts with a common response of encephalitis, and highest viral production in neurons.

The consensus that human infection with respiratory CoVs can cause encephalitis is supported by animal models, which have facilitated descriptions of this route of transmission. The infection begins in the airway epithelia, with binding of the virus to ACE2 receptors and subsequent alveolar involvement. Extrapulmonary viral particles are then spread to the CNS, possibly through nuclei that regulate the respiratory rhythm in the brain stem, or through the olfactory bulbs. The mechanism of neuroinvasion in respiratory CoVs may occur via neuronal contacts and the recruitment of axonal transport.

Although other neural cells expressing ACE2 and CD13 receptors are also susceptible, as neurons are the main targets of CoV neuroinvasion, the neuronal losses induced by regulated cell death are probably the main neurovirulence factor causing generalized encephalitis (14, 16). Such losses likely occur via mitochondria-associated caspase-independent apoptosis. However, glutamate homeostasis dysregulation and toxicity could be additional factors associated with brain damage, cardiorespiratory center inhibition, and microglial inflammatory reactions (32, 34–36).

The inflammatory processes involved in the CNS response to CoV infection remains unclear. While immune cell infiltration is weakly supported (24), the participation of astrocytes and other glial cells in these processes have been extensively reported. The expression of inflammatory cytokines and chemokines, together with an upregulation of their receptors, have revealed the role of microglial activation in the inflammatory reactions of the CNS (18, 23, 24, 28, 29).

The neurovirulence of respiratory CoVs appears to be associated with the duration of viral exposure, which is crucial for the severity of pathogenicity. The persistence of CoV may lead to a spectrum of conditions from acute encephalitis without noticeable sequelae, to paralysis or chronic disease, as illustrated by Lau et al. (21), Arbour et al. (17), Brison et al. (34), and Tseng et al. (29). The findings of those reports show that viral reactivation does not lead to increased inflammation, increased response of virus-specific T cells, or re-expression of cytolytic effector function. Virus-specific Tcells within the brain retain the ability to secrete viral antigen but are unable to prevent CNS viral reactivation.

While interferon-γ is crucial for viral clearance during acute infection, it is insufficient to control viral reactivation. Such inability to enhance T-cell effector function is attributed to the decreased ability of peripheral Tcells to access the CNS, and an inability of antigens to leave the CNS and reactivate peripheral Tcells (41, 42). These effects all lead to an absence of T cell-mediated cytokines in the brain (23) and an insufficiency of the Tcell-mediated immune response to CoVs in the CNS.

Several viruses can infect neural tissue cells and possibly participate in the induction of neurological diseases, even though their primary site of infection in humans may not be the CNS (43). For instance, neurodegenerative diseases, such as dementia-related disorders and/or sequelae might also be linked to viral infection. The association of CoVs with MS has been reported in some studies (18, 40) and immune cell virus cross-reactivity could be one of the pathological mechanisms by which it arises (30).

One notable point is the mutagenicity of CoVs. Even a couple mutations designed in the laboratory were able to increase the specificity of the virus to human CNS cells (22, 26, 34, 35, 38). This finding strongly supports an analogous process in SARS-CoV2 during the pandemic of Covid-19, through which we may be confronted with new neural-specific viral infections. Nevertheless, retrospective analysis of the data on CoV infections of the neural system could facilitate the prediction of viral behavior, indicate the weak points, and inform the prognosis of cases, within the context of future medical challenges.

### Limitations

Although previous findings on respiratory CoVs are useful for this Pandemic moment, more novel, in-depth studies on SARS-CoV2 are required to gain a better understanding of the events following the recent epidemics of Covid-19. All available studies this submission date referred to strains of CoV viruses that cause respiratory infections other than SARS-CoV2.

## Data Availability Statement

All datasets presented in this study are from articles publically available on the PubMed database (https://pubmed.ncbi.nlm.nih.gov/).

## Author Contributions

GA: conceptualization and data collection and curation; GA and EG: writing—original draft preparation; EM-C and PC: writing—review & editing; PC: supervision; PC and EM-C: project administration and funding acquisition. All authors contributed to the article and approved the submitted version.

## Conflict of Interest

The authors declare that the research was conducted in the absence of any commercial or financial relationships that could be construed as a potential conflict of interest.
